# Development of an Adapted Version of the Motor Competence Assessment (MCA) for Older Adults

**DOI:** 10.3390/jcm14217866

**Published:** 2025-11-05

**Authors:** Bruno Silva, Luís Paulo Rodrigues, Pedro Bezerra, José Maria Cancela Carral

**Affiliations:** 1 Instituto Politécnico de Viana do Castelo, Escola Superior de Desporto e Lazer, 4900-320 Melgaço, Portugal; silvabruno@esdl.ipvc.pt (B.S.); lprodrigues@esdl.ipvc.pt (L.P.R.); pbezerra@esdl.ipvc.pt (P.B.); 2 Sport Physical Activity and Health Research & Innovation Center (SPRINT), 4960-320 Melgaço, Portugal; 3 Faculty of Education and Sports Science, University of Vigo, 36005 Pontevedra, Spain; 4 HealthyFit Research Group, Galicia Sur Health Research Institute (IIS Galicia Sur), 36312 Pontevedra, Spain

**Keywords:** aging, physical function, functional independence, assessment adaptation

## Abstract

**Background/Objectives**: Age-related declines in motor and functional abilities can compromise independence and quality of life in later life. Motor competence (MC) plays an important role in maintaining quality of life and independence. However, few reliable instruments exist to assess MC in this population. The study adapts the Motor Competence Assessment (MCA) battery to meet the MC assessment and safety requirements of community-dwelling older adults. **Methods**: Seventy-six community-dwelling, physically active older adults (age = 73.4 ± 7.0 years) enrolled in a multi-phase adaptation process involving expert review, pilot and field testing, and validation of six motor tasks across three MC domains. Adaptations emphasized in the following four stages: accomplishing participant safety, autonomy, and the reliability of MC measurement principles. **Results**: The adapted version demonstrated very high completion rates, being safe and reliable for accessing MC, showing strong reliability in the manipulative domain. The use of the Challenge by Choice principle improved participant autonomy, confidence, and perceived motor competence. The main alterations to stability and locomotor tasks allow feasibility while maintaining test validity. Adjustments in instructions and practice trials reduced cognitive load and improved performance, addressing age-related perceptual and comprehension challenges. Ball Kicking and Throwing Velocity tests showed high reliability (ICC between 0.828 and 0.925), with minor gender-related differences. **Conclusions**: The adapted MCA is a safe, feasible, and reliable instrument for assessing MC in community-dwelling older adults. It preserves the conceptual foundations of MC while accommodating age-related MC alterations, offering a valuable resource for research and clinical applications.

## 1. Introduction

Biopsychosocial changes occur throughout the life span, often resulting in declines in functional and motor capacities [1,2]. However, these changes can be effectively managed and adapted to in older adulthood through the Selection, Optimization, and Compensation (SOC) framework proposed by Baltes and Baltes (1990) [3]. SOC strategies tend to develop progressively across the life course and may reach their peak during middle adulthood. Differences in goal orientation between younger and older adults can be attributed to variations in perceived and actual resource availability. These differences play a crucial role in mitigating functional decline and supporting autonomy in later life [4].

Physical function in older adults is a multidimensional construct shaped by habitual physical activity, body composition, and muscle capacity. Declines in movement efficiency, unfavorable shifts in the muscle-to-fat ratio, and reductions in strength and endurance collectively contribute to decreased mobility, heightened frailty, and an elevated risk of disability [5]. Additionally, age-related changes in brain function reveal altered patterns of neural recruitment, indicating a shift in motor control strategies with aging [1]. Older adults often show increased activation in prefrontal and parietal brain regions, suggesting a greater reliance on cognitive control mechanisms to offset declining motor efficiency. This compensatory neural activity implies that aging disrupts the automaticity of motor processes, necessitating additional cognitive resources to sustain performance. However, given the prefrontal cortex’s vulnerability to age-related decline, this strategy may become less effective over time, potentially leading to reduced motor adaptability and increased error rates during complex or demanding tasks [1].

Motor Competence (MC) refers to the development and performance of human movement, and it has been defined as a person’s ability to be proficient in a broad range of locomotor, stability, and manipulative gross motor skills [6,7]. It is a fundamental component of human development, influencing physical performance and overall functional capacity, facilitating the learning of new skills, and motor proficiency on novel motor tasks throughout the lifespan [8]. From a developmental perspective, MC follows a progressive trajectory, with fundamental motor skills emerging in infancy and becoming increasingly refined throughout childhood [9]. As a central component of motor development, MC is a key determinant of physical activity engagement and long-term movement proficiency [10,11]. The theoretical construct of MC encompasses three core domains of fundamental motor skills: locomotor (e.g., running, jumping), stabilizing (e.g., balance, postural control), and manipulative (e.g., object control, throwing) [12,13].

When considered alongside the Selection, Optimization, and Compensation (SOC) framework, the functional capacity of older adults, and age-related changes in neural recruitment patterns, MC appears to play a regulatory role in modulating adaptive behavior and sustaining motor performance across the lifespan. Therefore, assessing MC is essential, not only to identify age-related differences in motor performance but also to highlight the preserved potential for motor learning in later life. Moreover, MC assessment informs the development of evidence-based practices and interventions, ensuring that scientifically validated tools are used to support the health and well-being of older adults [14].

Despite its significance, there remains a prominent gap in the literature regarding MC assessment in older populations. Current assessments fall short in feasibility, fairness, sensitivity, and adaptability [15].

The Motor Competence Assessment (MCA) was developed to address this gap, offering a lifespan-representative tool that provides an alternative approach to evaluate the essential MC constructs [8,16].

Designed to be objective, reproducible, and efficient, the MCA comprises six tests that comprehensively assess all three fundamental motor skill domains: stability, locomotor, and manipulative skills [6,11], incorporating skills that do not demonstrate cultural specificity [15]. Developing a lifespan-spanning, culturally valid, and ecologically sensitive assessment tool could transform how we understand the role of motor competence in health and cognition across the human lifespan [15].

This study aims to advance the assessment and monitoring of motor competence in older adults by adapting the Motor Competence Assessment (MCA) to better address the specific needs of the aging population. The goal is to provide a more precise and relevant tool for evaluating motor competence, ultimately supporting strategies that enhance mobility, independence, and quality of life in later life.

## 2. Materials and Methods

This cross-sectional study aimed to adapt the Motor Competence Assessment (MCA) for older adults. A two-phase process was implemented, involving expert evaluation, feasibility testing with elderly participants, and experimental validation of motor tasks, particularly in tasks that involve higher risk or require the manipulation of balls that can have different sizes. The adaptation process was conducted with prior authorization from the instrument’s original authors [6,11]. The study was approved by the Technical-Scientific Commission—CTC-ESDL-CE006-2021, following the ethical guidelines outlined in the Helsinki Declaration. Before participation, all participants received a detailed information sheet outlining the study’s purpose, significance, procedures, and potential risks and benefits. Afterwards, provided written informed consent, ensuring they were fully informed and could voluntarily engage in the research.

### 2.1. Participants

A convenience sample of 76 physically active, community-dwelling autonomous older adults (22 males and 54 females), ages between 66 and 88 years (mean age = 73.36 ± 6.99), were recruited from a community-based program focusing on physical activity and exercise for older adults (Table 1).

Approximately one-third of participants reported having no formal education, while the majority had completed up to the 12th grade. A small percentage held a bachelor’s degree or higher. Regarding health conditions, most participants did not report having hypertension, high cholesterol, or diabetes. However, hypertension was prevalent among male participants. All individuals were physically active, engaging in exercise at least twice per week.

Data collection took place in a controlled laboratory environment to ensure standardized conditions and enhance the reliability and accuracy of the measurements. Inclusion criteria were 1. age over 65 years; 2. regular engagement in physical activity (e.g., structured exercise programs, community fitness classes, or recreational sports); 3. independence in activities of daily living; and the ability to ambulate without assistance. Exclusion criteria include the following: 1. age 65 years or younger; 2. lack of regular physical activity (e.g., no participation in structured exercise programs, community fitness classes, or recreational sports); and 3. inability to ambulate independently.

### 2.2. Anthropometric Assessment

Data were collected using standardized instruments to ensure accuracy and consistency. Height was measured with a SECA 217 stadiometer, and body mass was assessed using a SECA 813 scale, both calibrated according to manufacturer guidelines. Body mass index (BMI) was then calculated from these measurements.

### 2.3. Biosocial Assessment

To collect information on educational attainment, health conditions, and physical activity, participants answered simple and straightforward questions designed to facilitate accurate self-reporting and ensure reliable data collection. Participants indicated their highest level of formal education completed, selecting from categories ranging from no formal education to bachelor’s degree or higher. For health status, participants reported whether they had been diagnosed with hypertension, high cholesterol, or diabetes, responding “yes” or “no” to each. Physical activity was assessed by asking participants how many times per week they engaged in exercise activities related to the project or others.

### 2.4. Motor Competence Assessment

The original Motor Competence Assessment (MCA) [6,11] consists of six tests that assess three motor skill components: stability, locomotor, and manipulative. The Stability component is evaluated with the Lateral Jumps (LJ) and Shifting Platforms (SP) tests. In the LJ test, participants must jump sideways with both feet together as quickly as possible for 15 s, over a small wooden beam (60 cm length × 4 cm height × 2 cm width) positioned in the center of a rectangular area (100 cm length × 60 cm width). Correct jumps (those performed without touching outside the marked area or stepping on the beam) are counted, and the best score from two trials is recorded.

For the SP test, participants must move sideways between two wooden platforms (25 cm × 25 cm × 2 cm), reaching for the platform in the ground next to them, moving it to the opposite side, and stepping into it as quickly as possible for 20 s. Both feet have to be on the platform before moving to the other platform. Each complete movement cycle earns two points (one for shifting the platform and another for stepping onto it). The highest score from the two trials is recorded.

The locomotor component comprises the 10 m Shuttle Run (SHR) and the Standing Long Jump (SLJ). For the SHR, participants perform a 4 × 10 m shuttle run at maximal speed. Beginning behind a designated line, they sprint to the opposite line, retrieve a wooden block, and return to place it at the starting line. This process is repeated for a second block, and the final line is crossed, having the block in hand (no need to place it on the floor). The best time of the two trials is recorded in seconds and hundredths of a second. The SLJ is executed by performing a standing long jump for maximum distance. Starting with both feet on a marked line, each participant performs three jumps, with the longest distance measured from the starting line to the back of the heel at landing. The best result is recorded in centimeters.

The manipulative component includes ball throwing (BTV) and kicking velocity (BKV), with the size of the balls being age-dependent in the original MCA protocol. In this study, BTV participants are asked to throw a baseball (diameter: 7.3 cm; mass: 142 g) against a wall using an overarm action with preparatory balance. The BKV is performed with a standard soccer ball size 5 (circumference: 68 cm; mass: 410 g) against a wall with maximal force and preparatory balance. Peak velocity in meters per second (m/s) is measured using a radar gun system behind a designated target on the wall. Each participant performs three trials, with the highest velocity recorded as the final score.

### 2.5. Procedures

The Motor Competence Assessment (MCA) was originally developed to measure motor competence across the lifespan, with normative data currently available for individuals aged 3 to 23 years, providing normative data for children, adolescents, and young adults. The MCA is a practical, qualitative, and easy-to-administer battery that evaluates motor competence through a balanced set of six tests, two per component or subscale [6,11]. Although the adaptation and normative values for older adults are lacking, highlighting the need for careful adaptation of the MCA to evaluate motor competence and detect functional decline in healthy aging populations [6,11].

Furthermore, being designed for a broad age range, adapting the MCA for older adult populations is essential to detect functional decline within healthy aging, and for that, careful adaptations are necessary. The professionals involved in the adaptation and application of the tests in this study were two expert researchers with more than six years of experience in the application of the MCA with children and adolescents.

Four stages were outlined to implement the adaptation process of MCA for the elderly population. Stage 1 involved a preliminary pilot test conducted in a laboratory setting with two physically active, community-dwelling, autonomous elderly people (one man aged 68 and a woman aged 71), who were not previously familiar with the MCA or the researchers. The entire procedure, including the initial interview, test application, and data recording, was administered individually by the researchers. For the BTV and BKV, the adulthood standard was used (baseball and size 5 balls). The results were later reviewed and compared to confirm inter-observer agreement, serving as a baseline for feasibility and clarity of instructions.

In stage 2, real-world application and participant experience were tested. The tests were extended to a group of 10 physically active, community-dwelling, autonomous older adults (6 women, mean age = 72.83 ± 4.45, and 4 men, mean age = 75.00 ± 9.49) who participate in regular physical activity programs at least twice a week. On the day of the testing, informed consent and medical history were collected to ensure the safety of the participants by identifying any risks that could interfere with performance or increase the chance of injury during the assessment. The MCA was then administered, with one researcher leading the test administration and the other supporting the participants when necessary. Both researchers documented observations for each of the six tests, particularly focusing on difficulties and strategies used. All participants were encouraged to perform each task in accordance with the adapted protocol and relative to their individual capabilities. When a participant was unable to complete a valid repetition according to the primary test criteria, a score of zero was recorded, and the researcher allowed the participant to either discontinue the attempt voluntarily or continue until the test time elapsed. For example, in the Shifting Platforms test, if a participant was unable to grasp or move the platform correctly (e.g., dragging it on the floor), those attempts were excluded from scoring, and only valid repetitions were counted. This procedure ensured that all participants were evaluated under standardized and objective conditions, thereby minimizing bias and preserving the integrity of the data collection process.

Only completed tests without external help were considered valid. External help was defined as any physical assistance (e.g., touching, supporting, or directing the participant) that could influence performance. The adapted protocols did not constitute outside help, as they were integral to ensuring safe participation while maintaining task validity. Only verbal encouragement was allowed to motivate participants to complete the tasks safely and confidently.

In Stage 3, a modification and adaptation of the MCA tests was proposed, given a comprehensive analysis of the observation data and results collected in Stage 2, to identify common limitations, challenges, and difficulties experienced by the participants during the assessment. The assessment was performed sequentially, beginning with stability tasks, followed by locomotor tasks, and concluding with manipulative tasks. Challenges included difficulty understanding instructions, balance impairments, reduced proprioception and coordination, and movement limitations. All documented difficulties and limitations were reviewed by the two researchers. In cases of disagreement or unclear findings, the targeted literature review was conducted, or a senior expert in motor competence was consulted. It is important to highlight that the adaptations were not made due to the inability to perform the tasks, but rather to minimize the risk of false negatives in motor competence, ensuring that the test reflects true ability, not limitations caused by testing conditions or lack of age-appropriate adjustments and safety risks, such as falls. The proposed adaptations were then evaluated and approved by a panel of four experts in exercise, motor development, and aging, which included one of the original creators of the MCA. A specific emphasis was placed on the ball size used in the throwing and kicking tasks, with the aim of evaluating whether different ball sizes influenced performance outcomes.

In Stage 4, data were collected to calculate Intraclass Correlation Coefficients (ICC) to evaluate the reliability and consistency of performance using different ball sizes in the throwing and kicking tasks. This analysis was crucial to ensure that the adaptations maintained the integrity of the assessment and did not introduce variability due to equipment changes. Additionally, this stage aimed to validate the overall effectiveness and safety of all proposed adaptations by administering the final version of the battery to a larger sample of physically active older adults enrolled in structured exercise programs.

### 2.6. Statistical Analyses

Descriptive statistics were used to summarize the characteristics of the participants. In Stage 4, the intraclass correlation coefficient (ICC) based on a two-way mixed-effects, single measurement, consistency from ICC(3,1), for evaluating repeated measures by the same rates was used for reliability. Cronbach’s Alpha was additionally calculated to assess internal consistency when using different ball sizes for the throwing and kicking tasks. Systematic bias between ball versions was evaluated with Bland–Altman analyses according to all sample and stratified by sex. Mean difference and 95% limits of agreement were calculated. Reliability was not performed for stability and locomotor domains.

All analyses were performed using SPSS software version 28.0.1.0 for Mac (IBM, USA).

## 3. Results

The adaptation process of the Motor Competence Assessment (MCA) comprises four distinct stages. Each stage aimed to incrementally evaluate the feasibility, reliability, safety, and applicability of the battery among community-dwelling older adults who engage in regular physical exercise.

### 3.1. Stage One: Initial Pilot Test

Two older adult participants completed the original MCA protocol in a controlled laboratory setting. During the assessment, both participants showed hesitation, specifically in the Lateral Jumps (LJ) and Standing Long Jump (SLJ) tasks. In the LJ, the requirement for side-to-side movement, in the lateral plane, was noted as challenging, and the wooden beam used in the task was perceived as an increasing obstacle as time progressed. Both participants expressed difficulty with landing using both feet together in the LJ and SLJ tasks, reporting fear of falling during these landings. Overall, the protocol was well-tolerated by participants, and the tasks were achievable. However, instructions required clarification in several instances, as participants occasionally struggled to fully understand the original phrasing, and more step-by-step guidance was found to be more effective in facilitating understanding. All tasks were completed, but with visible caution and adjustments by the participants, especially in LJ and SLJ tasks.

### 3.2. Stage Two: Small-Scale Field Test

Ten community-dwelling older adults enrolled in a physical activity program were evaluated in the MCA within a community setting. Regarding the difficulties that emerged in Stage one, when performing the LJ and SLJ tasks, participants were permitted to absorb ground reaction forces by landing either with both feet together or one foot at a time. However, the starting position was consistent with both feet together, and the rest of the procedure remained unchanged. During the assessment, participants experienced similar difficulties in the LJ and SLJ tasks but were more confident when landing with one foot at a time. In the LJ task, one fall and two episodes of losing balance were reported after one foot hit the wooden beam. In the SLJ task, one fall was also reported after a participant landed with both feet together and was unable to maintain the position due to strength issues. Several participants showed difficulties with ball manipulation and kicking, potentially related to strength and/or coordination issues. Misunderstanding of the instructions was minimal, since the researchers demonstrated, explained, and allowed practice of the tasks during the warm-up for each task group (stability, locomotor, manipulative) before starting the tests. Most participants completed between five and six tasks independently. Adaptations were required for four participants during the SP task, allowing them to grab the platform with one hand and drag it along the floor. All safety incidents occurred without injuries or the need for medical intervention, thanks to the presence of the two researchers near the participants.

### 3.3. Stage Three: Expert Panel Review and Final Adaptation

Difficulties and limitations identified during Stage two, including challenges observed in the Lateral Jumps (LJ) and Standing Long Jump (SLJ) tasks, such as falls, loss of balance, and difficulties in ball manipulation and kicking, were thoroughly reviewed by the research team and subsequently discussed with the expert panel. Equipment dimensions, timing, and scoring systems are generally preserved, but some adaptations are proposed, namely the removal of the wooden beam in the LJ task, which was substituted with a tape strip of identical dimensions, thereby minimizing the physical barrier. Other adaptations were performed in the SP task, permitting participants to grasp the platform with only one hand. Due to difficulties in reaching a consensus regarding manipulative tasks, in Stage four, manipulative tasks will be performed using varying ball sizes, allowing the calculation of intraclass correlation coefficients and evaluation of reliability. The final version maintains the original structure, with two tests for each domain of motor competence (stability, locomotor, manipulative), and incorporates essential adaptations to ensure safety, feasibility, and respect for individual capacities in older adults, in line with the principle of “Challenge by Choice”, meaning participants decide their level of participation and effort. Allowing the participants to decide the extent to which they engaged in each task based on their comfort, confidence, and perceived ability emphasizes autonomy and respects individual differences in motor competence and risk tolerance. These factors indirectly reflect perceived motor competence through their task selections and engagement levels. This observational approach aligns with the understanding that perceived motor competence reflects an individual’s awareness and belief in their capability to perform motor tasks [17]. No formal instrument was used to measure perceived motor competence, participants’ engagement, task selection, and choices regarding effort or strategy. However, participants who chose stepping rather than jumping in SLJ or lifting the platform with one hand in the Shifting Platforms (SP) task were considered to be self-regulating their engagement in line with their perceived competence and safety considerations.

For the SP, the equipment, timing, and scoring method remained the same. Participants can pick up the platform with one or both hands, but it must not be dragged across the floor. However, if the platform is dragged across the floor, that repetition is not counted. This means that, by choice, the participant may use one or both hands to lift it from the ground. They must stand laterally beside the platform before stepping onto it. Observers should stay close to prevent a sudden loss of balance.

In the execution of the LJ, the time and scoring are maintained. The wooden beam is replaced by a 2 cm wide tape, maintaining all other dimensions. Participants may choose to jump over the tape or step one foot at a time, without touching the line or leaving the designated area, always joining feet before moving to the next repetition. If able and preferred, they can jump with feet together, and observers should always remain close for safety.

In the standing long jump, adaptations included that participants may choose to jump with both feet together, one foot at a time, or simply take the longest step possible. They must start with feet together and parallel, and land as far as possible on one or two feet. Distance is measured from the starting line to the closest heel at landing. If sliding occurs during landing, measurement is taken from the first heel contact. If unclear, the test should be repeated.

The original procedure and scoring are kept for the 10 m shuttle run. However, it is important to emphasize that participants do not have to run but are encouraged to move as fast as they can safely. Each participant should perform at least one practice trial to adjust their approach, grasp, and carrying of the block.

For the throwing and kicking velocity, the protocol is maintained. Participants first warm up and perform two or three low-speed practice throws and kicks with guidance to familiarize themselves with the movement. While no feedback is given on limb positioning during the execution, an explanation and practice of the supporting foot placement before the movement is provided. For these two tests, velocities were measured using a Bushnell Velocity Radar Gun (Bushnell Corporation, Overland Park, KS, USA). The radar was positioned approximately one meter behind the participant, aligned with the trajectory of the ball, and placed at a height to match the ball’s path during execution. A fixed target was placed on the wall to standardize an aim across trials, and the participants were placed behind a line at four meters from the wall. Participants performed attempts in a randomized order between ball types.

In general, administration must consider that observers should position themselves close to participants during all tests to prevent falls or sudden loss of balance; participants can decide how much to engage in each task and set their own goals (according to the “Challenge by Choice” principle); and practice trials are mandatory to allow participants to warm up, fully understand what is expected, and recognize safety strategies.

### 3.4. Stage Four: Validation with Broader Sample

In the validation experience of the proposed changes, over 90% of participants completed all tasks independently. Physical support was required in fewer than 10% of tasks, mainly in SP. No injuries or adverse incidents were reported, with the final version of the test assuming the adaptation presented in Table 2.

For the throwing velocity test (BTV), the overall ICC(3,1) was 0.955 (95% CI: 0.930–0.986, *p* < 0.001), and Cronbach’s Alpha was 0.977. These results indicate excellent internal consistency and high equivalence between the different versions of the balls used (baseball and tennis balls), suggesting that both are suitable for reliable measurement of the participants’ performance.

The ICC analysis for the ball kicking velocity (BKV), the overall ICC(3,1) was 0.902 (95% CI: 0.850–0.968, *p* < 0.001), and Cronbach’s Alpha was 0.949, indicating high reliability between the two ball versions analyzed (football number four or five).

Repeating the same analyses by gender showed some disparities between the results for men and women for the BKV. The female group had an ICC(3,1) of 0.828 (95% CI: 0.721–0.945, *p* < 0.001) and Cronbach’s Alpha of 0.906, indicating good reliability. The male group showed an ICC(3,1) of 0.872 (95% CI: 0.718–0.972, *p* < 0.001) and a Cronbach’s Alpha of 0.932, reflecting satisfactory reliability between test versions.

Regarding BKV, women showed an ICC(3,1) of 0.919 (95% CI: 0.865–0.976, *p* < 0.001), and Cronbach’s Alpha of 0.958, indicating high internal consistency, while the male group presented an ICC of 0.955 (95% CI: 0.892–0.991, *p* < 0.001) and a Cronbach’s Alpha of 0.977, demonstrating excellent reliability across test forms.

Bland–Altman analyses performed for the entire sample indicated minimal systematic bias between ball versions. For BKV, the mean difference was 0.12, with 95% limits of agreement from −2.85 to 3.08. For BTV, the mean difference was −0.49, with limits from −2.50 to 1.53. These results demonstrate good agreement between ball versions. Women, mean difference for BKV was 0.02 (95% limits of agreement: −2.84 to 2.89), and for BTV –0.47 (−2.61 to 1.66). For men, the mean difference for BKV was 0.35 (−2.86 to 3.57), and for BTV, −0.52 (−2.20 to 1.16), demonstrating good agreement and no relevant systematic bias between the different ball versions.

Reliability was confirmed for the manipulative tasks. However, they were not performed for the stability and locomotor domains due to safety considerations during assessment.

## 4. Discussion

This study effectively adapted the Motor Competence Assessment (MCA) battery to meet the capabilities and safety requirements of community-dwelling older adults engaged in regular physical activity. The findings align with the evidence that MCA have a lifespan perspective and evaluate multiple discrete skill domains [15], establishing the biopsychosocial frameworks that describe the decline in motor and functional capacities with aging [1,2]. However, consistent with the Selection, Optimization, and Compensation (SOC) model [3], older adults can adapt to these changes by selecting and optimizing motor strategies that allow them to maintain functional independence [4]. The stepwise adaptation process, including pilot testing, field testing, expert review, and broad validation, ensured that tasks remained feasible, reliable, and safe without compromising the integrity of the original protocol. Particularly, the incorporation of the “Challenge by Choice” principle empowered participants to tailor their engagement, promoting autonomy and minimizing risk [18]. These factors indirectly reflect perceived motor competence through their task selections and engagement levels, reflecting an individual’s awareness and belief in their capability to perform motor tasks [17].

The final adapted MCA demonstrated excellent reliability across tasks, with high completion rates and no reported injuries, confirming its suitability for this population. However, the adaptation process revealed specific challenges in tasks requiring dynamic balance and complex motor coordination, such as the Lateral Jumps (LJ) and Standing Long Jump (SLJ). Removing physical barriers, such as replacing the wooden beam with tape, and allowing the use of individual strategies for execution, such as stepping or jumping with one or both feet, effectively addressed these concerns. These adaptations are intended to be security and age-adjusted and not eliminate participants’ hesitancy and fear of falling, which reflects broader proficiency barriers that impact motor competence throughout the lifespan.

The ability of some participants to perform only one or no repetitions, as well as their fear of attempting certain tasks, underscores the real-world manifestation of these proficiency barriers, highlighting how motor competence evolves and may decline over time, influencing functional capacity and confidence [6,7,9].

The suggested adaptations, such as permitting alternative landing techniques and replacing physical obstacles with less threatening markers, exemplify how older adults utilize compensatory strategies to optimize performance while respecting safety, consistent with SOC principles [3] and the assessment of movement consistency, as well as the capability to successfully perform a goal with different movement coordination [15]. Moreover, the cognitive load associated with understanding and executing complex or unfamiliar motor tasks may be higher for older adults, particularly when instructions are not simplified. This was evident in early testing stages, where simplified instructions and practice trials significantly improved comprehension and performance, emphasizing the use of practice trials and clear, demonstrated instructions minimized misunderstandings, an essential consideration given the cognitive and sensory changes that can accompany aging [1]. These limitations are also indicative of declining motor skill proficiency and confidence, which can restrict participation in daily activities and increase the risk of functional decline. Recognizing and accommodating these barriers within assessments is crucial for obtaining a realistic understanding of motor competence and for developing tailored interventions that address both physical and psychological components of motor performance [10,19]. Ball kicking velocity (BKV) and ball throwing velocity (BTV) tests showed to be reliable measures across different ball sizes, demonstrating excellent internal consistency and no relevant systematic bias between the different ball versions. The slight gender differences observed, where males demonstrated marginally higher agreement, do not compromise the overall reliability but may reflect small variations in measurement stability between male and female participants.

The observation in Stage one that participants consistently began the LJ and SLJ tasks with both feet together reflects adherence to standardized starting procedures. However, the variation in landing, particularly in Stage two, suggests participants adapted their landing strategy, possibly to enhance stability or comfort. This flexibility in landing technique likely represents an instinctive adjustment to manage balance or reduce perceived difficulty, while maintaining the integrity of the initial task requirements. The distinction between a fixed starting position and variable landing highlights how participants negotiate task demands within established constraints, demonstrating motor adaptability in older adults [14], and the consistency of the adaptation for the elderly. These results support the MCA battery as a valid, reliable, and feasible tool for assessing motor competence in older adults.

These adaptations preserved the assessment’s objectives while enhancing safety and participant confidence, addressing a critical gap in the literature [15]. Its comprehensive coverage of fundamental motor skill domains and incorporation of safety and autonomy principles, such as the “Challenge by Choice” approach, makes it a valuable resource, since this autonomy-driven model respects individual differences in physical capacity and motivation, which is critical in older populations with varying health statuses [20]. These adaptations also offer a practical and reliable tool for assessing motor competence among older adults in community and clinical settings, allowing the opportunity to assist as a useful screening instrument to identify individuals at risk of mobility decline or falls and to tailor interventions accordingly. Although in this validation process, and to ensure safety, autonomy, and adaptation to MC alterations due to aging, particularly in the stability and locomotor tasks, e.g., use of tape instead of a beam, option to step instead of jump, or controlled movement pace, may influence direct comparability with the normative values established for children and young adults. Consequently, the current results should be interpreted as reflecting an adapted version rather than as scores directly comparable with the 3–23-year-old MCA. Future research should focus on establishing these age and sex specific reference values and establishing appropriate cut-off points. Still, future research should also focus on validating the battery’s predictive ability for adverse outcomes such as falls or functional decline and exploring longitudinal changes in motor competence over time. Additionally, expanding testing to diverse populations and settings will strengthen the generalizability of findings, taking into consideration the success of its use and adaptation to people with intellectual disability [21] and children with atypical development [22].

Despite its strengths, this study had some limitations. First, the initial stages involved relatively small sample sizes. Second, the fact of testing only physically active community-dwelling older adults does not allow a full perspective and generalization across broader populations of older adults, including those with varying health conditions and levels of physical activity. Third, the adaptation process focused on accommodating physical limitations and safety concerns but did not extensively address the potential influence of cognitive impairments common in older populations. Addressing these limitations in future research and developing longitudinal studies to establish the clinical relevance and utility of the battery in monitoring aging trajectories will strengthen the evidence base for MCA use and contribute to more effective motor competence assessment and intervention strategies for older adults.

## 5. Conclusions

The proposed adapted Motor Competence Assessment battery is a reliable, safe, and feasible tool tailored for older adults, emphasizing participant autonomy and safety without compromising measurement quality. Therefore, the findings primarily reflect community-dwelling physically active older adults, and caution is warranted when extrapolating to more fragile or sedentary individuals. However, safety-driven procedural changes in stability and locomotor tasks should be considered an adapted version that may require independent normative values. Its implementation could enhance motor competence evaluation throughout the lifespan and contribute to better-informed interventions aimed at maintaining functional independence and quality of life in aging populations.

## Figures and Tables

**Table 1:** Sample characterization.

Variable	Category/Statistic	Total (n = 76)	Female (n = 54)	Male (n = 22)
Age (years)	mean ± SD	73.36 ± 6.99	73.27 ± 7.18	73.60 ± 6.66
BMI (kg/m^2^)	mean ± SD	27.75 ± 4.59	27.70 ± 4.93	27.88 ± 3.63
Height (m)	mean ± SD	1.59 ± 0.09	1.56 ± 0.07	1.68 ± 0.05
Body Mass (kg)	mean ± SD	69.77 ± 11.39	66.48 ± 10.13	78.66 ± 9.95
Educational Attainment	No education	30.7%	32.1%	27.3%
	4th grade	14.7%	15.1%	13.6%
	9th grade	20.0%	20.8%	18.2%
	12th grade	26.7%	22.6%	36.4%
	Bachelor’s/Degree	8.0%	9.4%	4.5%
Hypertension	Yes	45.7%	42.3%	55.6%
	No	54.3%	57.7%	44.4%
Cholesterol	Yes	46.1%	55.6%	22.7%
	No	39.5%	35.2%	50.0%
Diabetes	Yes	24.6%	24.5%	25.0%
	No	75.4%	75.5%	75.0%

mean ± SD, where SD = standard deviation; BMI = Body Mass Index; m = meters; kg/m^2^ = kilograms per square meter; kg = kilograms.

**Table 2:** Motor Competence Assessment (MCA) final adaptations for the elderly.

Test	Original	Adaptations for Older Adults	Safety and Support Measures	Participant Autonomy
Shifting Platforms	Move sideways between two 25 × 25 × 2 cm platforms for 20 s, lifting with both hands without dragging; 1 point for moving the platform, 1 point for stepping onto it; each complete transfer = 2 points.	Equipment, timing, and scoring unchanged. Participants may lift the platform with one or both hands, but it must not be dragged. Participants have to be laterally positioned to the platform and execute laterally.	Observer positioned nearby to prevent falls or loss of balance.	“Challenge by Choice”—participants decide how to perform the lift.
Lateral Jumps	Jump sideways with feet together over a 60 × 4×2 cm beam for 15 s on a 100 × 60 cm surface; one point per correct jump without stepping on the beam or outside the rectangle; best score recorded.	Original timing and scoring maintained. Wooden beam replaced with a 2 cm wide tape. Participants may step over the line one foot at a time or jump with feet together or alternated, ensuring feet are joined before each repetition. Must stay within the designated area.	Close monitoring to respond to balance issues.	“Challenge by Choice”—jump or step depending on ability and preference.
Standing Long Jump	Jump forward with feet together; measure distance from starting line to back of the heel at landing; longest distance recorded.	Participants may jump with both feet, one foot at a time, or step forward as far as possible. Start with feet together and parallel. Distance measured to closest heel. Repeat if landing is unclear.	Observer stands nearby for safety during takeoff and landing.	“Challenge by Choice”—choose how to jump or step.
10-Meter Shuttle Run	Run 4 × 10 m at maximal speed following an acoustic signal: start at the starting line, run to the 10 m line to pick up the first of two blocks placed there, return and place it across the starting line; run back to the 10 m line, pick up the second block, and return across the starting line to finish the test. Time stops when the second block crosses the starting line.	Procedure and scoring unchanged. Emphasis that running is not required. Participants should move as fast as safely possible. A practice trial is required to familiarize with approach, grasp, and block carrying.	Clear demonstration and a practice run. Observer ensures safety during transitions.	“Challenge by Choice”—participants choose pace and effort.
Ball Throwing Velocity	Throw a standard tennis or baseball (according to age) against a wall at maximum speed using an overarm throw with preparatory balance.	Warm-up and 2–3 low-speed practice throws allowed. No feedback on technique, but guidance on spatial orientation is provided. Standard baseball ball.	Adequate space and warm-up. No performance correction given.	“Challenge by Choice”—decide intensity and attempt style.
Ball Kicking Velocity	Kick standard soccer ball size 4 or 5 (according to age) against a wall at maximum speed using a preparatory balance.	Warm-up and 2–3 practice kicks at low speed. No technical feedback during execution, but explanation and practice of support foot placement are provided. Standard soccer ball size 5.	Adequate space and warm-up. No performance correction given.	“Challenge by Choice”—participants control effort and execution.

## Data Availability

The datasets generated and analyzed during the current study are not publicly available but are available from the corresponding author on reasonable request.

## Abbreviations

The following abbreviations are used in this manuscript:

MC	Motor Competence
MCA	Motor Competence Assessment
